# Central arch reconstruction and thoracic endovascular aortic repair for complicated acute type B aortic dissection with aberrant right subclavian artery

**DOI:** 10.1016/j.xjtc.2021.09.029

**Published:** 2021-09-17

**Authors:** Andy Dong, William D. Jordan, Bradley G. Leshnower

**Affiliations:** a Division of Cardiothoracic Surgery, Department of Surgery, Emory University School of Medicine, Atlanta, Ga; b Division of Vascular and Endovascular Surgery, Department of Surgery, Emory University School of Medicine, Atlanta, Ga

**Figure:**
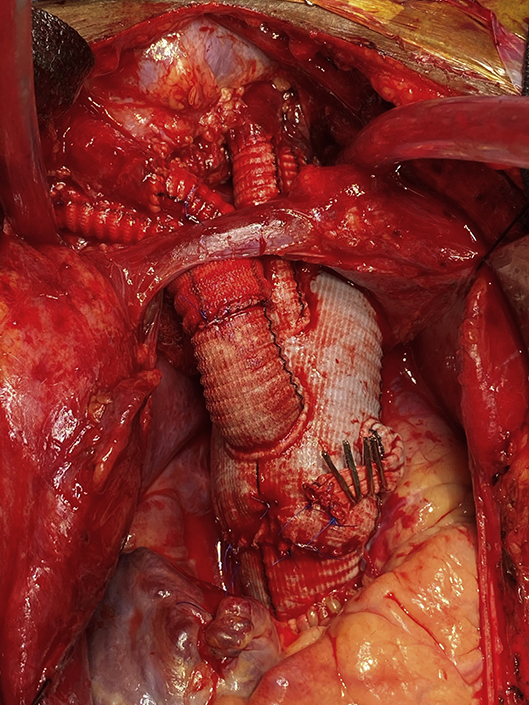
Aortic arch reconstruction with multibranch graft and anastomoses to supra-aortic vessels.

**Central Message**

Central 4-vessel aortic arch reconstruction with TEVAR is a feasible strategy in treating complicated acute type B aortic dissection with aberrant right subclavian artery.

See Commentary on page 181.

Aberrant right subclavian artery (ARSA) is a rare anatomic variant in which the right subclavian artery originates distal to the left subclavian artery (LSA) and crosses the midline in a retroesophageal course before assuming its right subclavicular position. Patients with acute type B aortic dissection (ATBAD) and ARSA pose a unique challenge due to the absence of an appropriate proximal landing zone for thoracic endovascular aortic repair (TEVAR) without covering both subclavian arteries. In this case series, 2 different hybrid techniques of central 4-vessel arch reconstruction with TEVAR are described to treat 4 patients with complicated ATBAD and ARSA.

## Technique #1

A 49-year-old female patient presented with ATBAD and ARSA with a 5-cm Kommerell diverticulum and right iliofemoral malperfusion. The primary intimal tear (PIT) originated distal to the LSA, and the dissection flap extended into the ARSA and distally to the iliac arteries. Given the lack of an adequate proximal landing zone and the presence of a 4-cm ascending aorta, single-stage ascending aortic replacement, 4-vessel arch debranching, and a zone 0 TEVAR was recommended.

Following hemisternotomy, the patient was placed on cardiopulmonary bypass (CPB) via right axillary artery cannulation and right atrial venous cannulation. Ascending aortic replacement and debranching of the right and left carotid arteries and LSA were performed using a customized multibranched graft without the use of hypothermic circulatory arrest. After the patient was weaned from CPB, the ARSA was transected and ligated proximally and distally (due to persistent dissection) at the level of the right clavicular head, and a neoascending aorto-right axillary bypass was constructed via the right pleural space.

**Figure 1:**
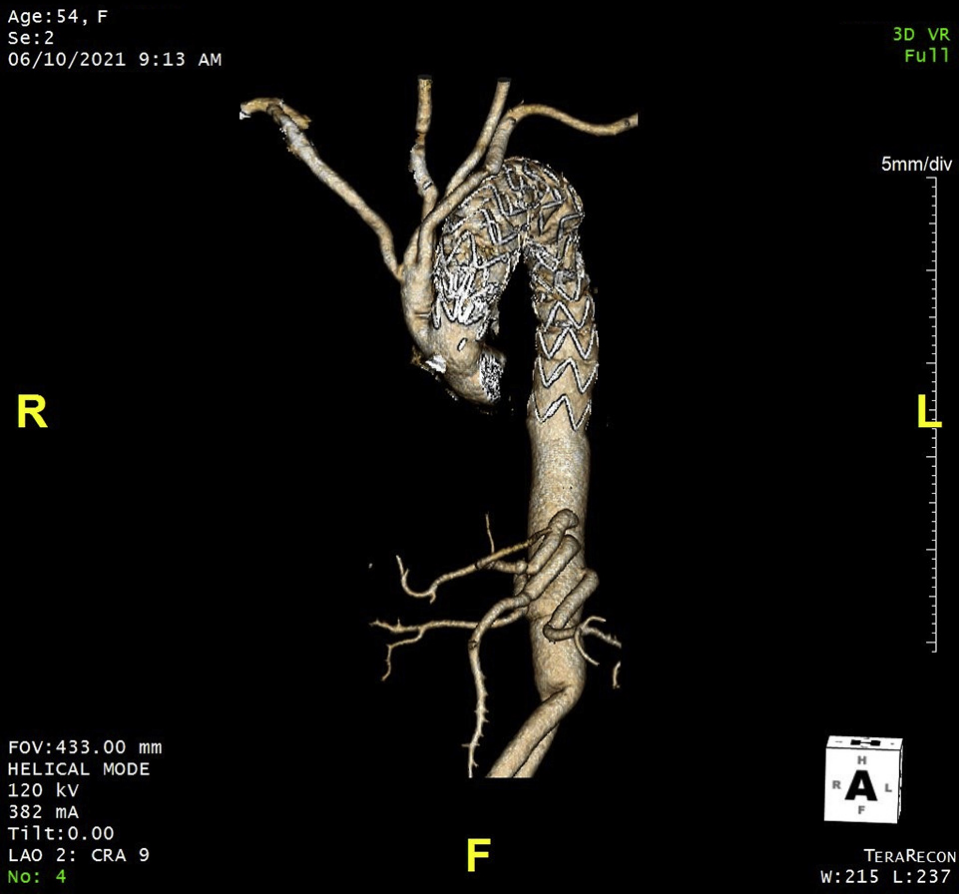
Three-dimensional computed tomography after 4-vessel arch debranching and zone 0 thoracic endovascular aortic repair (technique #1) and open replacement of the distal descending aorta to bilateral common iliac arteries.

Once arch reconstruction was complete, a transfemoral zone 0 TEVAR was performed with endograft coverage extending to the celiac artery, which resolved the iliofemoral malperfusion. The patient had an uneventful postoperative course and was discharged home. Surveillance imaging demonstrated complete obliteration of the thoracic false lumen with reconstitution of the false lumen in the abdominal aorta. On surveillance imaging 4 years after her index procedure, she had developed a 6.0-cm extent IV thoracoabdominal aortic aneurysm. She subsequently underwent an open repair using left heart bypass, with aortic replacement from the distal descending to the bilateral common iliac arteries (Figure 1).

## Technique #2

Three patients underwent treatment of their complicated ATBAD with ARSA using a frozen elephant trunk/central 4-vessel reconstruction procedure. The PIT in 2 patients originated at the ostium of the LSA, and the PIT in the third patient originated in a 5.5-cm Kommerell diverticulum. All patients had an inadequate proximal landing zone for TEVAR and underwent the procedure described to follow.

**Figure 2:**
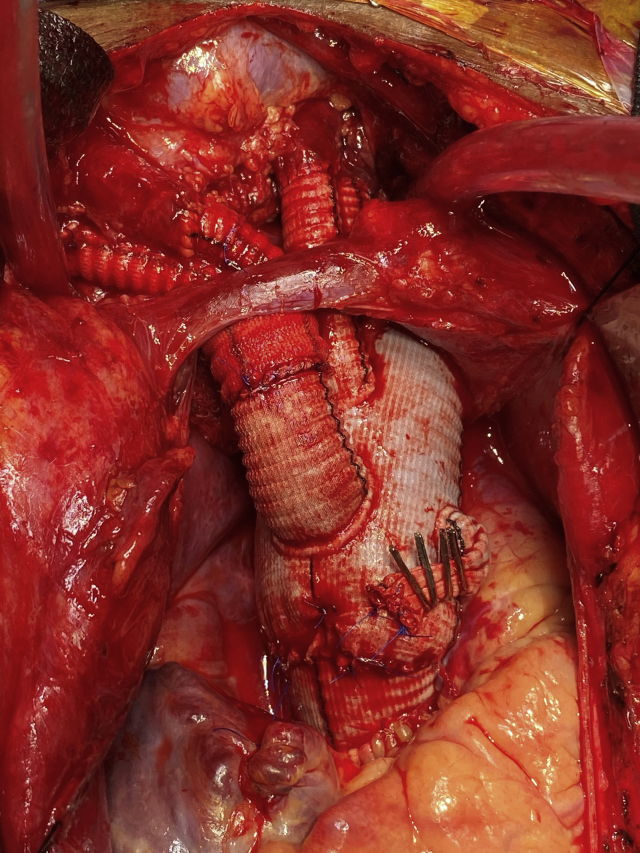
Intraoperative image of the proximal aorta after aortic arch reconstruction with a multibranch arch graft and anastomoses to the native supra-aortic vessels (technique #2).

Under intravascular ultrasound guidance via a femoral sheath, a Benson wire was placed into the true lumen and advanced into the ascending aorta, where it was left for TEVAR deployment later in the case. Next, a sternotomy was performed and the ARSA was isolated at the level of the right clavicular head, transected, and ligated proximally. An 8-mm graft was anastomosed to the distal end of the ARSA and clamped for reattachment later to the neo-innominate artery. Central aortic and right atrial cannulation was used to initiate CPB, and during the cooling period, the right carotid artery was transected, oversewn proximally, and an 8-mm graft was anastomosed to the distal right carotid artery and connected to a second arterial limb of the CPB circuit. Once the bladder temperature reached 25°C, hypothermic circulatory arrest was initiated with unilateral antegrade cerebral perfusion via the right carotid artery. After arch excision, antegrade TEVAR was performed over the Benson wire, and the distal anastomosis incorporating the stent graft was performed in zone 3 using a multibranched arch prosthesis. CPB was reinstituted, and the LSA and left carotid artery were individually reimplanted, followed by the proximal aortic anastomosis and crossclamp removal. The ARSA and right carotid artery were reimplanted to a 14 × 7-mm aortobifemoral graft that had been previously anastomosed to the 14-mm neo-innominate artery branch of the multibranched arch graft (Figure 2).

All patients had uncomplicated postoperative courses and were discharged home. Surveillance imaging demonstrated patency of all supra-aortic vessels, total obliteration of the thoracic false lumen, false lumen reconstitution in the abdominal aorta, and no aneurysmal degeneration of the abdominal aorta.

This study was approved by IRB00022795 (last updated March 8, 2021). Informed written consent was obtained from each patient to include their information in this publication in accordance with the principles set forth in the Helsinki Declaration.

## Comment

TEVAR is the gold standard for complicated ATBAD. However, patients with ARSA lack an adequate proximal landing zone for a multitude of reasons, including (1) proximity of the LSA and ARSA, (2) the presence of a Kommerell diverticulum, and (3) arch angulation. Approaching these patients via sternotomy enables central reconstruction of all supra-aortic vessels and the ability to either create a landing zone or, in the case of arch replacement, obviates the need for one.

The alternative approach to the aforementioned techniques is a staged bilateral carotid-subclavian bypass and TEVAR. However, the use of extra-anatomic cervical bypasses carries a risk of stroke, thoracic duct injury, recurrent laryngeal (5%) and axillary nerve injury (2%), and a reported 25% risk of phrenic nerve injury.^1^ Furthermore, this approach is associated with a high incidence of type IA endoleak and the risk of retrograde type A dissection.^2^ Single-stage central 4-vessel reconstruction with ascending and/or arch replacement essentially eliminates the risks of retrograde type A dissection, Ia endoleaks, and phrenic nerve injury. In summary, the aforementioned cases demonstrate that these 2 techniques are safe options in the treatment of complicated ATBAD with ARSA.
